# ChAdOx1 interacts with CAR and PF4 with implications for thrombosis with thrombocytopenia syndrome

**DOI:** 10.1126/sciadv.abl8213

**Published:** 2021-12-01

**Authors:** Alexander T. Baker, Ryan J. Boyd, Daipayan Sarkar, Alicia Teijeira-Crespo, Chun Kit Chan, Emily Bates, Kasim Waraich, John Vant, Eric Wilson, Chloe D. Truong, Magdalena Lipka-Lloyd, Petra Fromme, Josh Vermaas, Dewight Williams, LeeAnn Machiesky, Meike Heurich, Bolni M. Nagalo, Lynda Coughlan, Scott Umlauf, Po-Lin Chiu, Pierre J. Rizkallah, Taylor S. Cohen, Alan L. Parker, Abhishek Singharoy, Mitesh J. Borad

**Affiliations:** 1 Division of Hematology and Medical Oncology, Mayo Clinic, Scottsdale, AZ 85054, USA.; 2 Department of Molecular Medicine, Mayo Clinic, Rochester, MN 55905, USA.; 3 Center for Individualized Medicine, Mayo Clinic, Rochester, MN 55905, USA.; 4 Mayo Clinic Cancer Center, Phoenix, AZ 85054, USA.; 5 Biodesign Center for Applied Structural Discovery, Arizona State University, Tempe, AZ 85281, USA.; 6 Division of Cancer and Genetics, School of Medicine, Cardiff University, Cardiff CF14 4XN, UK.; 7 School of Molecular Sciences, Arizona State University, Tempe, AZ 85251, USA.; 8 MSU-DOE Plant Research Laboratory, Michigan State University, East Lansing, MI 48824.; 9 Department of Biochemistry and Molecular Biology, Michigan State University, East Lansing, MI 48824.; 10 Computational Structural Biology and Molecular Biophysics, Beckman institute, University of Illinois, IL 61801, USA.; 11 Institute of Infection Immunity and Inflammation, MRC-University of Glasgow Centre for Virus Research, Glasgow G61 1QH, UK.; 12 Medicines Discovery Institute, School of Biosciences, Cardiff University, Cardiff CF10 3AT, UK.; 13 Eyring Materials Center, Arizona State University, Tempe, AZ 85281, USA.; 14 Analytical Sciences, Biopharmaceutical Development, AstraZeneca, Gaithersburg, MD 20878, USA.; 15 School of Pharmacy and Pharmaceutical Science, College of Biomedical and Life Sciences, Cardiff University, Cardiff CF10 3NB, UK.; 16 Department of Microbiology and Immunology, University of Maryland School of Medicine, 685 W. Baltimore Street, MD 21201, USA.; 17 Center for Vaccine Development and Global Health, University of Maryland School of Medicine, 685 W. Baltimore Street, MD 21201, USA.; 18 Division of Infection and Immunity, School of Medicine, Cardiff University, Cardiff CF14 4XN, UK.; 19 Microbial Sciences, Biopharmaceuticals R&D, AstraZeneca, Gaithersburg, MD 20878, USA.

## Abstract

Vaccines derived from chimpanzee adenovirus Y25 (ChAdOx1), human adenovirus type 26 (HAdV-D26), and human adenovirus type 5 (HAdV-C5) are critical in combatting the severe acute respiratory coronavirus 2 (SARS-CoV-2) pandemic. As part of the largest vaccination campaign in history, ultrarare side effects not seen in phase 3 trials, including thrombosis with thrombocytopenia syndrome (TTS), a rare condition resembling heparin-induced thrombocytopenia (HIT), have been observed. This study demonstrates that all three adenoviruses deployed as vaccination vectors versus SARS-CoV-2 bind to platelet factor 4 (PF4), a protein implicated in the pathogenesis of HIT. We have determined the structure of the ChAdOx1 viral vector and used it in state-of-the-art computational simulations to demonstrate an electrostatic interaction mechanism with PF4, which was confirmed experimentally by surface plasmon resonance. These data confirm that PF4 is capable of forming stable complexes with clinically relevant adenoviruses, an important step in unraveling the mechanisms underlying TTS.

## Teaser

We observe previously unknown interactions between clinically important adenovirus vector capsids, platelet factor 4, and CAR.

## INTRODUCTION

The ChAdOx1 viral vector, adapted from chimpanzee adenovirus Y25 (ChAd-Y25), is the basis for the ChAdOx1 nCoV-19 vaccine (AZD1222/Vaxzevria) (*1*). ChAdOx1 nCoV-19 induces robust immunity against the severe acute respiratory coronavirus 2 (SARS-CoV-2), protecting against severe symptoms requiring hospitalization, in 100% of clinical trial recipients, and infection of any severity, in approximately 70% (*2*, *3*). A potentially life-threatening clotting disorder, thrombosis with thrombocytopenia syndrome (TTS), which presents similarly to heparin-induced thrombocytopenia (HIT) has been observed in a minority of AZD1222 recipients following the first but not the second dose (*4*–*6*). Similar observations have been made in recipients of the Janssen HAdV-D26.COV2.S vaccine, derived from the species D human adenovirus type 26 (HAdV-D26) (*5*, *7*). The pathological mechanism underpinning TTS is unknown, although recent reports highlight a probable role for platelet factor 4 (PF4) (*8*, *9*).

Detailed mechanistic understanding of the virus/host interactions of adenovirus-derived vectors has facilitated their advancement to the clinic. Previous work has shown that the presence of preexisting neutralizing antibodies targeting an adenoviral vector can limit therapeutic efficacy, neutralizing the vector before it has therapeutic effect (*10*, *11*). Following intravenous administration of human adenovirus type 5 (HAdV-C5), studies uncovered important in vivo interactions including high-affinity interactions with coagulation factor X (FX) and/or platelets. These interactions contribute to vector degradation and, consequently, to reduced therapeutic index (*12*–*14*). Collectively, this knowledge drove the field to develop adenoviral vectors with low seroprevalence, including ChAdOx1 and HAdV-D26, and engineering of capsid proteins to overcome these issues (*15*).

It is critical to investigate the vector-host interactions of ChAdOx1 to determine how it may contribute to rare adverse events like TTS. Here, we characterized the capsid structure of ChAdOx1 and the primary receptor binding fiber knob protein. We used this structural information to investigate its ability to interact with potential partners, including CD46, coxsackie and adenovirus receptor (CAR), and PF4. We confirmed our observations in vitro using cell-based experiments and surface plasmon resonance (SPR). These data clarify our understanding of whether ChAdOx1 interacts with host proteins thought to be involved in immunogenicity, HIT, and TTS.

## RESULTS

### The structure of the ChAdOx1/ChAd-Y25 viral capsid

**Fig. 1.**
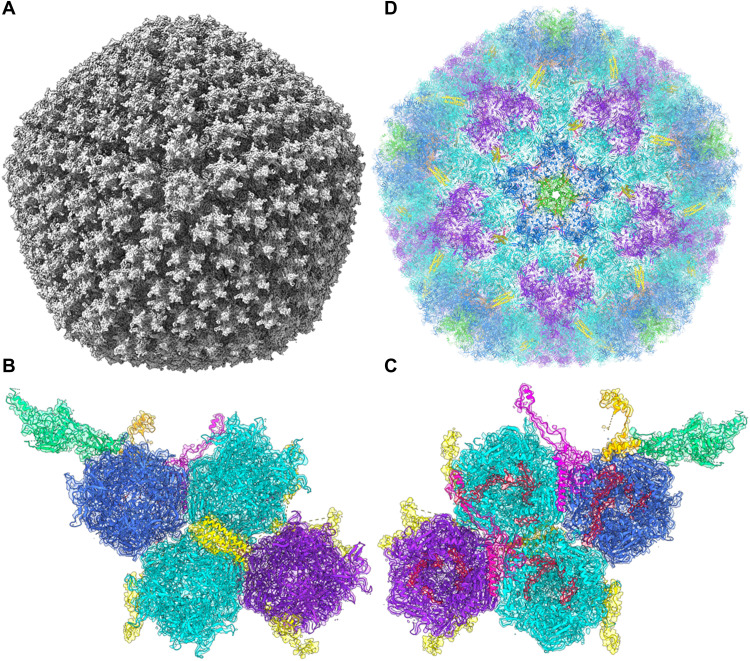
Capsid structure of the ChAdOx1 viral vector. Cryo-EM volume data for ChAdOx1 show an archetypical adenovirus icosahedron (**A**). This volume resolved to show an asymmetric unit containing one penton copy (green), one trimeric peripentonal hexon (blue), two 2′ hexons (cyan), one 3′ hexon (purple), a four-helix bundle corresponding to four copies of pIX (yellow), a peripentonal pVIII (magenta), a 2′ pVIII (pink), partial density for a pIIIa protein (orange), and six copies of pVI (red), seen from the capsid exterior (**B**) and interior (**C**) in their associated volume. Repeating these asymmetric units with T25 icosahedral symmetry enables the reconstruction of a full ChAdOx1 capsid model (**D**).

Determined at 3.07-Å resolution, ChAdOx1 has the quintessential icosahedral adenovirus capsid structure (Fig. 1A). As in other adenovirus structures solved by single-particle cryo–electron microscopy (cryo-EM), local resolution is higher on the more ordered interior of the capsid while the flexible components on the capsids’ exterior result in less resolved signal (fig. S1). The asymmetric unit contains the expected penton monomer; one peripentonal, two secondary, and one tertiary hexon trimers; one peripentonal and one secondary copy of Protein VIII; and partial density for the pIIIa protein (Fig. 1, B and C).

The pentameric penton protein, located at the 12 fivefold icosahedral vertices, had weaker signal (Fig. 1, B and C), which could indicate a less well-ordered, or less stably interacting, protein than those observed in previous cryo-EM structures of HAdV-D26 and HAdV-C5. As in other reported structures, the penton RGD loop (residues 305 to 335), which is responsible for binding to integrins following attachment to the cell surface via the fiber knob protein, was left unmodeled because of the lack of signal, indicative of its flexibility.

pVIII (Fig. 1C) was well resolved, barring residues 103 to 163, for which we observed no signal (Fig. 1E), in line with the observations of other groups (*16*, *17*). Partial density was observed for pIIIa, concentrated near the base of the penton (Fig. 1C). We observed partial densities for 12 copies of pVI in the base of the hexons that adopted two conformations, either wrapping around the hexon interior before extending into the capsid core or extending directly out of the base of the hexon to wrap over the top of pVIII (Fig. 1C). Hexon was well resolved, including the hypervariable regions (HVRs) on the exterior (Fig. 1C), enabling a complete reconstruction. Reconstruction of the full capsid atomic model results in minimal clashes between asymmetric units and reconstructs a full icosahedral capsid (Fig. 1D). The trimeric fiber protein, consisting of a long flexible shaft terminating in the globular knob domain, was not modeled as only some poorly resolved portions of the shaft were visible, likely due to its flexibility (fig. S1). Instead, the fiber knob was solved by crystallography.

### CAR is a high affinity ChAdOx1 fiber knob receptor

**Fig. 2.**
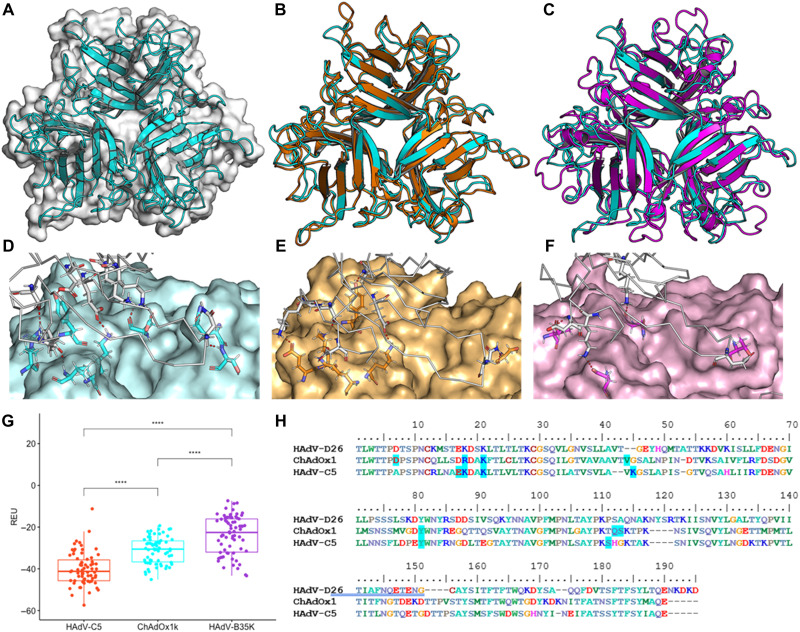
ChAdOx1 and HAdV-C5 have tight homology and form CAR contacts. The crystallographic structure of the ChAdOx1 fiber knob (cyan) shows the archetypical homotrimer (**A**) and aligns closely to the HAdV-C5 fiber knob (orange) with an RMSD of 1.4 Å (**B**) but does not closely align to the HAdV-B35 fiber knob [purple (**C**)]. Homology models equilibrated by molecular dynamics show ChAdOx1 (**D**), and HAdV-C5 fiber knobs (**E**) form numerous polar contacts (red dashes) with CAR (white ribbon), although HAdV-B35 (**F**) forms few. Free energy calculations in Rosetta show that HAdV-C5 forms the strongest predicted interaction with CAR, followed by ChAdOx1, and then HAdV-B35 (**G**). Mapping the predicted contact residues (blue highlight) in HAdV-C5 and ChAdOx1 to the clustalω aligned sequences shows similar contact residues are conserved in HAdV-D26 (**H**). ****P < 0.001. REU, Rosetta Energy Units.

Adenovirus fiber knob is responsible for the primary virus-cell interaction during infection. To investigate primary ChAdOx1 receptors, we solved the structure of the ChAdOx1 fiber knob receptor (Fig. 2A). Data from one crystal were used for the final structure analysis (fig. S2A), showing P1 symmetry with cell dimensions: *a* = 98.427 Å, *b* = 112.26 Å, *c* = 98.605 Å, and β = 92.6°. Diffraction data were scaled and merged at 1.59-Å resolution. The fiber knob shows the expected three monomers assembling into a homotrimer with threefold symmetry (Fig. 2A), packed into an asymmetric unit containing four trimeric copies (fig. S2B). The electron density map was of sufficient quality to determine side-chain conformations throughout most of the structure (fig. S2, C and D).

Superpositioning of the previously reported structure of HAdV-C5 fiber knob and our structure of the ChAdOx1 fiber knob protein show that fold homology is high, despite only 64.86% amino acid sequence homology, including in the loops, with a root mean square deviation of 1.4 Å (Fig. 2B). There is much lower homology between ChAdOx1 and HAdV-B35 fiber knob. The core fold is similar, but the loops are divergent (Fig. 2C).

Using the structure of HAdV-D37 fiber knob in complex with CAR as a template, we generated models of the ChAdOx1, HAdV-C5 (a CAR-interacting adenovirus), and HAdV-B35 (a CD46-interacting adenovirus, which does not bind CAR) fiber knobs, in complex with CAR (fig. S3, A to C). ChAdOx1 and HAdV-C5 were both predicted to form numerous polar contacts with CAR (Fig. 2, D and E), with HAdV-B35 forming very few (Fig. 2F). Rosetta interface energy calculations supported these observations, suggesting that the strongest CAR interaction was formed by HAdV-C5, followed by ChAdOx1, and then HAdV-B35 fiber knob (Fig. 2G). These calculations are in line with previous observations (*18*). Mapping interacting residues back to the ChAdOx1 sequence shows that several predicted CAR-binding residues are shared with HAdV-D26 and HAdV-C5 fiber knobs (Fig. 2H).

**Fig. 3.**
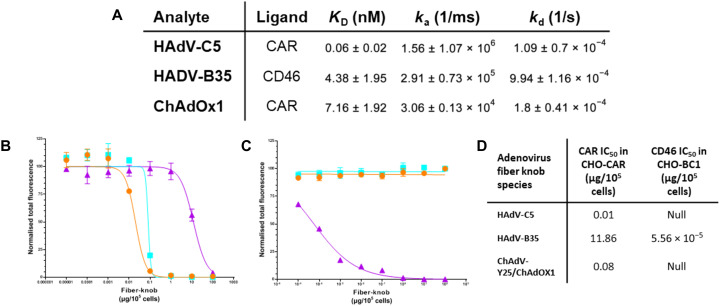
ChAdOx1 fiber knob binds to CAR as a high affinity receptor. SPR experiments demonstrated HAdV-C5 and ChAdOx1 form high affinity interactions with CAR, and HAdV-B35 forms high affinity interactions with CD46 (**A**). Antibody inhibition experiments on CHO-CAR (**B**) and (CD46-BC1 isoform expressing) CHO-BC1 (**C**) cells show HAdV-C5 (orange) and ChAdOx1 (cyan) bind CAR with high affinity but not CD46, while HAdV-B35 (purple) binds to CAR with very weak affinity and CD46 with high affinity (**D**).

To validate these predicted CAR interactions, we performed SPR of ChAdOx1, HAdV-C5, and HAdV-B35 fiber knob proteins binding CAR or CD46. This confirmed that HAdV-C5 and ChAdOx1 fiber knobs bind strongly to CAR but not CD46. HAdV-B35 binds strongly to CD46. HAdV-C5 remains the strongest known CAR-binding adenovirus with an equilibrium binding constant (*K*_D_) of 0.06 nM, with ChAdOx1 binding at 7.16 nM, not as strong as HAdV-C5 due to a slower on rate (*K*_a_). These results are summarized in Fig. 3A. SPR traces are in fig. S4 (A to C). We assessed whether these three fiber knobs bind CD46 and desmoglein 2 (DSG2). ChAdOx1 formed a weak interaction with CD46 with very fast on (*k*_a_) and off (*k*_d_) kinetics (fig. S4E). We observed a weak interaction between HAdV-B35 and CAR, likely indicative of nonspecific incidental interactions (fig. S4F).

We validated these finding in a cellular context using antibody inhibition assays in CHO (Chinese Hamster Ovary)–CAR cells, which express CAR but are negative for other known primary adenovirus receptors. HAdV-C5 showed that the strongest CAR-binding affinity [lowest median inhibitory concentration (IC_50_)] followed by ChAdOx1.HAdV-B35 demonstrated weak CAR-binding affinity (IC_50_ > 1000× higher) (Fig. 3B). It is possible that very high HAdV-B35 fiber knob protein concentrations give a false signal via nonspecific CAR interactions in both this and the SPR experiments. A similar analysis in CHO-BC1 cells, expressing CD46-BC1 isoform but no other known primary adenovirus receptors, determined that HAdV-B35, had a strong CD46 interaction, while HAdV-C5and ChAdOx1 fiber knobs did not have a measurable ability to prevent anti-CD46 antibody binding (Fig. 3C). These experiments, summarized in Fig. 3D, are robust evidence that ChAdOx1 uses CAR as a primary receptor, not CD46 or DSG2.

### Charge complementarity facilitates a ChAdOx1 complex with PF4

**Fig. 4.**
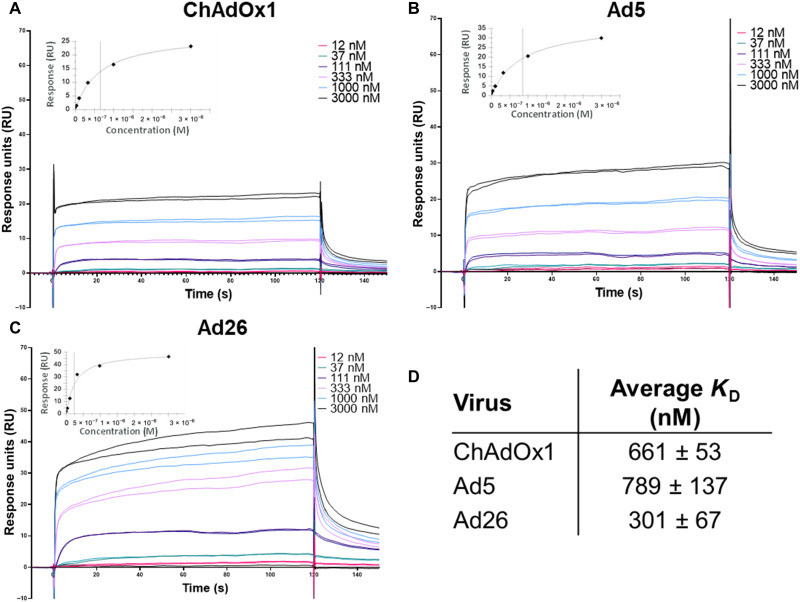
Clinical adenoviruses bind to PF4 with nanomolar affinity. Single-injection SPR experiments show that ChAdOx1 (**A**), HAdV-C5 (**B**), and HAdV-D26 (**C**) form stable and reproducible interactions with PF4. Affinity was calculated using the steady state model (**D**), and the curve showed close fit to the tested concentrations (insets).

Recent reports indicate that ChAdOx1 may interact with PF4, which is involved in HIT that has a similar clinical presentation to TTS (*8*). We used SPR to investigate whether PF4 can interact with highly pure preparations of adenovirus-derived vaccine vectors Ad26, Ad5, and ChAdOx1 or to the vaccine preparation of ChAdOx1 and AZD1222. ChAdOx1, Ad5, and Ad26 were observed to bind to PF4 with affinities of 661, 789, and 301 nM, respectively (Fig. 4). AZD1222 was observed to have a similar affinity to PF4 as the purified virus counterpart, with an affinity of 514 nM (fig. S5).

**Fig. 5.**
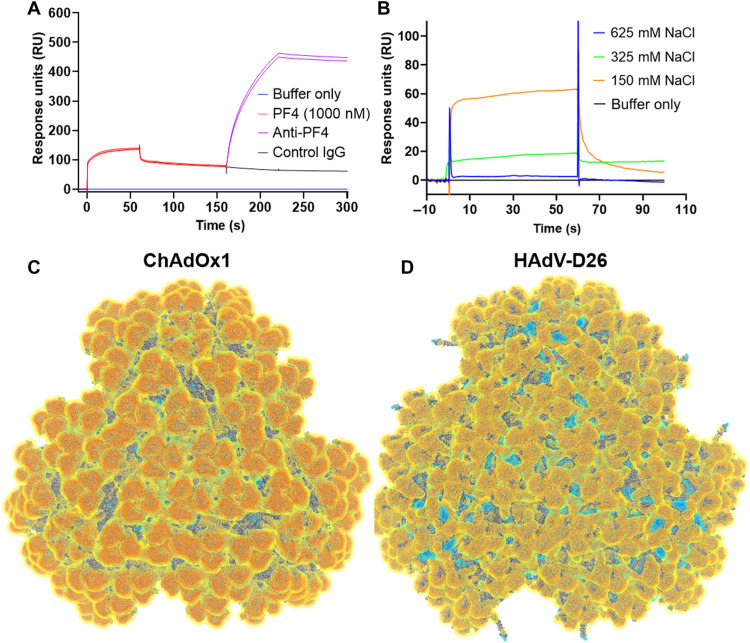
ChAdOx1 creates a stable complex with PF4 and is highly electronegative. A two-injection SPR experiment showed that, following PF4 binding to the ChAdOx1 capsid, a polyclonal αPF4 was able to bind to the surface of the chip indicating a ChAdOx1/PF4/antibody complex (**A**). IgG, immunoglobulin G. Similar SPR experiments show decreasing PF4 binding at increasing concentrations of NaCl (**B**). Visualization of the capsid (three vertices shown) of ChAdOx1 (**C**) and Ad26 (**D**) shows the electrostatic potential at −0.5 *k*_B_*T* (yellow), −1.0 *k*_B_*T* (orange), −1.5 *k*_B_*T* (red), 0.5 *k*_B_*T* (cyan), 1.0 *k*_B_*T* (blue), and 1.5 *k*_B_*T* (dark blue). Electronegative potential is focused at the apex of the hexons. ChAdOx1 is more electronegative than HAdV-D26 with negative charge extending into the interhexon spaces, while in HAdV-D26, it has electropositive charge in these regions. A more detailed capsid view is available in fig. S6.

Specificity of PF4 binding was confirmed by anti-PF4 antibody binding to the ChAdOx1/PF4 complex on the chip (Fig. 5A). This demonstrates the ability of antibodies to bind to PF4 while it remains in complex with ChAdOx1. We repeated the SPR experiments using different salt concentrations and observed a reduced PF4 binding with increasing salt, suggesting an electrostatic interaction (Fig. 5B).

Continuum electrostatic surface potential calculations show that the capsid of ChAdOx1 has an electronegative surface potential of <−1.5 *k*_B_*T* (Boltzmann constant) across approximately 90% of its surface, interrupted in interhexon spaces occupied by pIX, where the surface potential rises to >−0.5 *k*_B_*T* (Fig. 5C and fig. S6A). We compared the surface potential to that of HAdV-D26, which is also implicated in TTS. HAdV-D26 has an overall electronegative surface potential but less strong than ChAdOx1 at <−1.0 *k*_B_*T*. In contrast to ChAdOx1, we observe regions of positive potential, up to >+1.5 *k*_B_*T*, recessed in the interhexon spaces (Fig. 5D and fig. S6B).

In both viruses, the positive potential is driven by apical regions of adenovirus’ major capsid protein, hexon, which we calculated to be most negative in ChAdOx1, followed by HAdV-C5, and then HAdV-D26 (fig. S6C). We also observed that PF4 has a strong electropositive surface potential (fig. S6D). These observations are consistent with an electrostatic mechanism of interaction between ChAdOx1 and PF4.

### PF4 binds to ChAdOx1 in the interhexon space

**Fig. 6.**
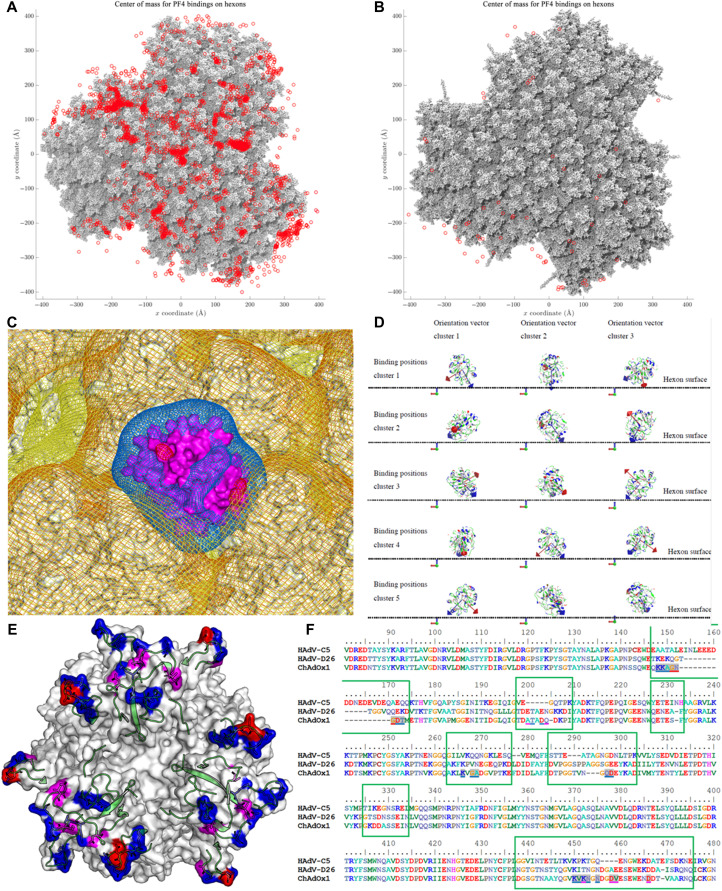
PF4 binds to ChAdOx1 in the interhexon spaces more frequently than it binds to Ad26. BD simulations of PF4 in solution with the facet show the locations at which PF4 makes contact with the facet (red spots) of ChAdOx1 (**A**) and Ad26 (**B**), showing that the most common interaction locus is the space between three hexons, where the PF4 (purple) is observed to sink into the space between hexons exposing electropositive regions to the electronegative hexons (**C**). Analysis of PF4-binding events shows that PF4 always forms contacts with ChAdOx1 oriented with its longest axis most normal to the plane of the hexon (**D**). Certain hexon residues are more commonly involved in the PF4 interaction (**E**), red residues interact >50% of the time, magenta residues interact >20%, and blue residues interact 20-1%. All these residues are within the HVR loops (green cartoon). These residues are underlined in the sequence alignment with Ad26 and Ad5 contained in the green boxes indicating the HVR sequences (**F**). Charge map coloring is the same as in Fig. 5.

To investigate a potential binding mechanism, we performed Brownian dynamics (BD) simulations of PF4 with the ChAdOx1 or HAdV-D26 capsid structure. Results show freely diffusing PF4 frequently contacts the capsid surface of ChAdOx1 between the hexons, most commonly at the interfaces of three hexons where negative charge is concentrated, and the interhexon space is maximized (Fig. 6A). HAdV-D26 also forms contacts with PF4 but less frequently (Fig. 6B). These simulations represent that possible binding initiation events should not be used to interpret relative affinity of interactions.

Analysis of an exemplar ChAdOx1/PF4-binding event at the threefold hexon interface shows the electropositive faces of PF4, occurring along its longest axis, oriented to face the electronegative potential of the surrounding hexons (Fig. 6C). To assess whether this orientation was a requirement for binding, we performed a clustering analysis of the contacts in our simulation. The analysis indicates PF4 is similarly orientated to the example in Fig. 6C in all contact instances (Fig. 6D). This common binding pose supports the idea that the ChAdOx1/PF4 interaction has specific determinants.

To identify amino acids that are potentially important for the ChAdOx1/PF4 interaction, we calculated the frequency of contacts between PF4 and the ChAdOx1 hexons in BD simulations. These indicate PF4 contacts residues in the HVRs of ChAdOx1 (Fig. 6, E and F). The HVRs face into the space between the hexons and are highly flexible, creating a fluctuating volume in the interhexon space (fig. S7, A and B).

### ChAdOx1/PF4 complex formation is inhibited by heparin

**Fig. 7.**
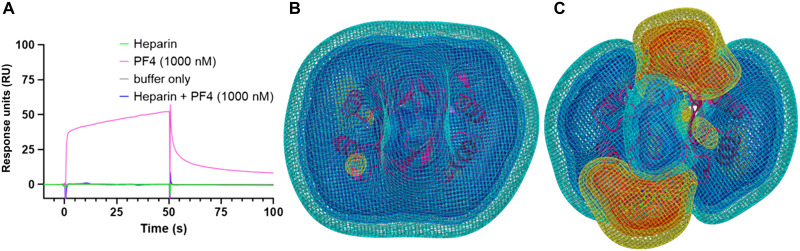
The ChAdOx1/PF4 complex is inhibited by the presence of heparin. SPR showed that preincubation of PF4 with heparin inhibits ability of PF4 to bind to ChAdOx1 (**A**). Calculations show that the highly electropositive charge on PF4 [purple (**B**)] is disrupted in the presence of heparin as shown by the increase in electronegative potential when PF4 is complexed by fondaparinux, a heparin-derived pentasaccharide (**C**). Charge map coloring is the same as Fig. 5.

Heparin is also a key component in the mechanism for HIT, binding to multiple copies of PF4 and forming aggregates with anti-PF4 antibodies that stimulate platelet activation via Fcγ Receptor IIa (RIIa). We performed SPR and BD experiments to test what effect heparin binding to PF4 has on the ability of PF4 to bind ChAdOx1. In BD experiments, we observed that PF4 in complex with fondaparinux, an anticoagulant drug composed of the PF4-binding heparin pentasaccharide, significantly reduced the number of binding initiation events with the ChAdOx1 capsid surface (fig. S8, A to C). In purified ChAdOx1 and the AZD1222 vaccine preparation, we observed that PF4 binding to ChAdOx1 was strongly inhibited by preincubation with heparin (Fig. 7A and fig. S8D). Continuum electrostatic calculations of PF4 and PF4 in complex with fondaparinux show that the electronegative potential of PF4 is ablated in the presence of fondaparinux (Fig. 7, B and C). We infer that this weakened electropositive potential may reduce the ability of PF4 to form an electrostatic association with ChAdOx1.

## DISCUSSION

We have resolved the capsid structure of the ChAdOx1 viral vaccine vector to a resolution of 3.07 Å, the highest reported resolution of an adenovirus capsid to date. We have characterized its primary cell attachment protein, the fiber knob (Figs. 1 and 2), and show that ChAdOx1, a simian adenovirus, shares considerable structural homology with human adenoviruses. We used our structural understanding and computational models to predict interactions between ChAdOx1 and host proteins. CAR is implicated in the transduction of host cells and is likely to be important for vaccine function. The other, PF4, contributes to pathogenesis in HIT and is hypothesized to be involved in TTS.

We confirm that CAR is a high-affinity receptor for ChAdOx1 fiber knob protein (Figs. 2 and 3). Given the established ability of ChAdOx1 to infect human and chimpanzee cells, it follows that it uses a receptor that is conserved between these species. CAR is an example of this as human and chimpanzee CAR proteins have identical amino acid sequences (fig. S9) (*1*). This is a verified example of human and primate adenoviruses sharing a common adenovirus receptor protein, although cross-species utilization of CAR has been observed previously as human and canine adenoviruses both use CAR as a high-affinity cell entry receptor (*19*, *20*).

We demonstrate that ChAdOx1 fiber knob does not bind CD46 or DSG2 (Fig. 3 and fig. S4). However, this does not preclude additional receptors. For example, HAdV-D37 and HAdV-D26 fiber knob proteins bind to both CAR and sialic acid–bearing glycans (*21*, *22*). Conceivably, there could be other mechanisms of direct interaction between the cell and capsid surface, such as the hexon-CD46 interaction, recently suggested for HAdV-D56, although it is unclear whether this putative interaction would be sufficient for a productive infection in vivo (*23*).

CAR, predominantly expressed in epithelial tissues, is involved in junctional adhesion (*24*, *25*). It has also been observed on the surface of human platelets, especially platelet aggregates and erythrocytes (*26*, *27*). Therefore, it may be tempting to try to link the affinity of ChAdOx1 for CAR with platelet aggregation and TTS. However, previous studies demonstrate that adenovirus-platelet aggregates are rapidly trafficked to the liver where they are sequestered by Kupffer cells and degraded (*28*). While it was possible to induce thrombocytopenia in mice following an intravenous dose of 10^11^ VP (Viral Particles)/ml replication-deficient adenoviral particles (*12*) (a dose ~7500× higher than that given, intramuscularly, in the ChAdOx1 nCoV-19 vaccine, assuming a 20-g mouse and a 75-kg human), this did not result in any thrombotic events. Another study in Rhesus macaques observed the opposite effect, longer clotting times, presumably as a result of the diminished platelet count and/or depletion of circulating FX (*29*, *30*). Therefore, we believe it is unlikely that direct association between ChAdOx1 and platelets drives thrombotic events, regardless of their CAR expression status, in TTS.

This study demonstrates binding between PF4 and ChAdOx1 under in vitro conditions, an interaction first suggested by Greinacher *et al*. (*31*), and presents a mechanism by which it could occur. SPR experiments demonstrate that PF4 binds, with nanomolar affinity, to highly pure CsCl gradient preparations of ChAdOx1 and the commercial ChAdOx1 nCoV-19 vaccine, AZD1222/Vaxzevria (Fig. 5 and fig. S5). This confirms that the association between Vaxzevria and PF4 is an interaction between the PF4 and ChAdOx1, rather than any cell line–derived proteins remaining in the vaccine following manufacture. We demonstrate that this interaction is not specific to ChAdOx1 and that PF4 forms interactions with Ad5 and Ad26 with similar affinity (Fig. 5). We also observed that heparin reduces the ability of PF4 to associate with ChAdOx1, suggesting that this interaction is not stimulated by PF4/polyanion complexes (Fig. 6 and fig. S8), which is concomitant with the finding that patients have not been treated with heparin before developing TTS (*4*).

Exploring the mechanism of this interaction, we demonstrate that PF4 frequently contacts the surface of ChAdOx1 when allowed to diffuse freely in BD simulations (Fig. 7A) and that these interactions are facilitated by electrostatic complementarity between the electropositive PF4 and electronegative ChAdOx1 capsid, matched with a shape complementarity that enables PF4 to enter the space between hexons (Figs. 6 and 7 and fig. S6). The electrostatic nature of this interaction was further supported by SPR experiments in the presence of increasing salt concentrations (Fig. 5B). Amino acid contacts are most frequent in the HVR loops (Fig. 7, E and F). Presently, it is unclear whether the contacts represent a specific amino acid interaction or are a function of the flexible HVRs entering the interhexon space where PF4 most commonly binds (fig. S7).

Ad26 has also been implicated in TTS at a similar frequency to ChAdOx1 on a per dose basis (*7*, *32*). Using a previously published model of Ad26 (*16*), we performed simulations as for ChAdOx1 and observed that PF4 contacted Ad26 less frequently than ChAdOx1 (Fig. 6, A and B). However, it is important to acknowledge that BD does not account for flexibility of the proteins following the initial interaction and operates on an accelerated time scale. Therefore, no inferences can be made regarding the protein’s residency times and whether a stable complex is formed. This should be explored in future molecular dynamics simulations.

Current evidence indicates that TTS presents similarly to HIT, a condition where patients present with blood clots following administration of the thromboprophylactic drug, heparin (*33*). This condition appears to be driven by anti-PF4 autoantibodies of sufficient affinity to cluster PF4 and create a multivalent, presumably higher avidity, interaction between the antibody Fc domains and FcγRIIa on the platelet surface, stimulating the platelet to release additional PF4. In the context of heparin, PF4 undergoes a conformational change, facilitating the binding of more common, lower affinity antibodies specific to the PF4-polyanion complex. This creates a positive feedback loop as antibodies bind to increasing copies of PF4, stimulating further platelet activation, culminating in the activation of the clotting cascade. This mechanism is described in detail by Nguyen *et al.* (*34*). To further summarize, whether anti-PF4 autoantibodies induce thrombosis, or not, is a function of concentration and antibody affinity for PF4.

Recent case reports show that most patients presenting with TTS (>90%) tested positive for αPF4 antibodies; however, incomplete medical history limits understanding of predisposing factors (*4*). Unlike those observed in HIT, anti-PF4 antibodies observed in TTS patients were predominantly of the subgroup that could bind to PF4 alone, rather than the PF4-heparin complex (*31*).

A ChAdOx1/PF4 complex could induce anti-PF4 autoantibodies. In this potential mechanism, small quantities of ChAdOx1 enter the blood through minor capillary injuries caused by the intramuscular injection, as has previously been observed (*35*). A ChAdOx1/PF4 complex could then form (Figs. 5 to 7), either independently or in association with platelets (*36*), and travel to the lymphatic system, transported by monocytes. Alternatively, PF4 released at the site of injection may complex the vector and drain directly to the lymphatic system. These virus/PF4 complexes may stimulate preexisting anti-PF4 memory B cells to differentiate into plasma cells, secreting anti-PF4 antibodies, which generally takes 4 to 8 days (*37*). This hypothesis is summarized in fig. S10. It is notable that TTS is much less frequently observed following the second dose of ChAdOx1, suggesting that, as in HIT, αPF4 immunoglobulin G is not long lasting and that any plausible mechanism should be prominent in the first but not second dose (*6*, *38*). Studies are needed to confirm whether adenovirus/PF4 complexes can induce thrombosis in the presence of anti-PF4 antibodies in vivo. This proposal goes some way toward explaining why TTS is observed so rarely, because it may require a series of low frequency stochastic interactions, first between small numbers of adenovirus particles entering the blood/lymph and then monocytes and/or B cells, which may only occur in individuals who are predisposed toward the generation of anti-PF4 antibodies.

In contrast to a previous proposal, we do not suggest EDTA, or residual HEK293 proteins, in the vaccine formulation contribute to TTS (*8*). EDTA is a component of the ChAdOx1 nCoV-19 vaccine formulation but is not present in the Ad26.SARS2.S vaccine, following which TTS has also been reported (*5*, *7*, *8*, *39*). We note that TTS symptoms manifest 5 to 24 days following vaccination, corresponding to the time line for a secondary antibody response (*8*). If large immune complexes formed directly with components of the vaccine formulation, then it seems more likely that they would lead to platelet activation and clot formation immediately following vaccination, rather than >5 days later. The discussion surrounding potential mechanisms for TTS has recently been reviewed (*9*).

Current clinical guidance from the World Health Organization advises against the use of heparin in the treatment of TTS, presumably based on the similar clinical presentation of TTS and HIT (*40*). Although our data suggest that heparin may inhibit the proposed interaction between ChAdOx1 and PF4, it does not provide any insights as to the effect of heparin on patients after they develop symptoms or its behavior in the wider biological context. Therefore, it is important to continue to adhere to current clinical guidance pending further studies on the role of heparin in TTS.

The ChAdOx1/PF4 interaction described in this study suggests potential mechanisms by which safer viral vectors might be engineered by ablating this interaction. “HVR swaps” have been performed with the goal of reducing recognition of adenovirus vector by neutralizing antibodies (*11*, *41*). Similar rational capsid engineering could eliminate electronegative residues in the HVRs, although a threshold below which the electronegative charge needs to be reduced has yet to be determined. Alternatively, upon determining key binding residues, a more specific approach could be envisaged where critical amino acids forming contacts with PF4 are removed or substituted. Therefore, modification of the ChAdOx1 hexon HVRs to reduce their electronegativity may solve two problems simultaneously: reduce the propensity to cause TTS and reduce the levels of antivector immunity, thus helping to maximize the opportunity to induce robust immune responses. Further exploration of adenovirus phylogenetic diversity may yield novel vectors with lower PF4-binding propensity and altered safety profiles.

Now, no proposed mechanism for TTS following vaccination is consistent with all the observed data. This is partly due to an incomplete clinical picture, a consequence of its rarity, consequently weak statistical data from which to draw inferences, and a lack of understanding about this novel interaction. Future work will focus upon clarifying whether the adenovirus/PF4 complex is inherently thrombogenic and, if so, what downstream interactions lead to this.

## MATERIALS AND METHODS

### Propagation of ChAdOx1 virus

T225 CellBind cell culture flasks (10×; Corning) were seeded with approximately 5 × 10^6^ human embryonic kidney 293 (HEK-293) T-REx cells (Invitrogen) each. Cells were cultured in Dulbecco’s modified Eagle’s medium (Gibco) supplemented with 10% heat-inactivated fetal bovine serum and 1% penicillin/streptomycin (Gibco) until 80% confluent. Cells were infected with ChAdOx1.eGFP, provided by the Coughlan Lab (University of Maryland), at a multiplicity of infection of 0.01. Cells were then monitored for cytopathic effect (CPE). When culture medium turned yellow, medium was replaced. Once CPE became evident, but the cells were not ready to be collected, yellowed medium was supplemented with sodium bicarbonate buffer (pH 7.4, Gibco) until it reddened. This was done to retain the virus released into the medium. Once CPE was observed in >80% of the cell monolayer, the cells (5 to 8 days after infection) were dissociated from the flask by knocking. The supernatant and cells were separated by centrifugation at 300*g* for 5 min, both were stored at −80°C until ready for purification.

### ChAdOx1 purification

ChAdOx1-containing medium was clarified by centrifugation at 4000 rpm for 10 min in a benchtop centrifuge. Supernatant was then loaded into 38-ml ultraclear tubes compatible with the SW28 rotor (Beckman Coulter) and centrifuged at 100,000*g* for 1 hour in a Beckman Coulter Optima XPN-100 ultracentrifuge. Supernatant was discarded, and the pellet, which was slightly yellow and sticky, was resuspended in 5 ml of phosphate-buffered saline (PBS) (pH 7.4, Gibco). This 5 ml of PBS containing the ChAdOx1 from the supernatant was used to resuspend the infected HEK-293 T-REx pellet. This solution was then mixed in a 1:1 ratio with tetrachloroethylene (TCE; Sigma-Aldrich) and shaken violently to ensure thorough mixing. The virus, PBS, and TCE mixture was then centrifuged at 2000 rpm in a benchtop centrifuge for 20 min. The top aqueous layer of the solution was removed by pipetting and placed into a new tube. A second TCE extraction was then performed by adding a further 5 ml of TCE to the aqueous layer, shaking, and centrifuging, as before. This is performed to ensure maximum removal cell debris. Previous purifications that excluded this second extraction showed fatty deposits when analyzed by negative stain transmission electron microscopy and resulted in viral aggregation. Next, the top aqueous layer was extracted again, and the remainder of the purification was performed using the two-step CsCl gradient method, as previous described (*42*), except for the following modification: During the final extraction of the virus containing band, which should have a crisp white appearance, the band was not removed using a needle through the side of the tube but by pipetting. A P1000 pipette was used to slowly withdraw fluid from the tube, taking from the meniscus, careful not to disrupt the band. As much fluid was removed above the band as possible, then a new pipette tip was used to withdraw the band from the meniscus in as little volume as possible.

This band was loaded into a 0.5-ml Slide-A-Lyzer cassette with a 100,000 MWCO (Molecular weight cut-off) (Thermo Fisher Scientific) and dialyzed against 1 liter of plunge freezing buffer [150 mM NaCl, 1 mM MgCl_2_, 20 mM tris (pH7.4)]. This solution was used to prepare grids for cryo-EM.

### Cryo-EM grid preparation

An UltrAuFoil grid (688-300-AU, Ted Pella Inc.) was glow-discharged on a PELCO easiGlow (Ted Pella Inc.) for 30 s. A Vitrobot Mark IV automated plunge freezer (Thermo Fisher Scientific) was used for blotting and freezing grids in liquid ethane using the following protocol: The sample chamber was set to 25°C and 100% humidity, 2.5 μl of sample was applied onto both sides of the grid, and a blot force of 1 and a draining time of 3 s were used for plunge freezing. The frozen grid was transferred to cryo-boxes under liquid nitrogen for storage.

### Cryo-EM data collection

The vitrified specimen was images using an FEI Titan Krios transmission electron microscope (Thermo Fisher Scientific) operating at an accelerating voltage of 300 keV with a Gatan K2 Summit direct electron detector camera (Pleasanton, CA) at a nominal magnification of ×37,313, corresponding to a pixel size of 1.34 Å/pixel at the specimen level. A total of 2875 movies, each consisting of 40 frames over the course of 8 s, were collected in superresolution mode with a subpixel size of 0.67 Å per pixel. Defocus setting was cycled from −0.5 to −1.6 μm for each exposure. The dose rate was adjusted to 2.59 e^−^ per pixel per second with 1.18 e^−^/Å^2^ per movie frame. Data were recorded and packed as a 4-bit and LZW-compressed TIFF format.

### Cryo-EM image processing and structure determination

Datasets were processed using RELION (version 3.1.1) (*43*) on a workstation with an Intel I7-6800K 3.4-GHz processor, 800 gigabytes of memory, a 1-terabyte SSD (Solid State Drive) drive, and 4 NVIDIA GTX 1080 GPUs (Graphic Processing Units). A large swap partition was set to 512 gigabyte. Motion correction was performed using RELION with a binning factor of 2 and removing the first three frames. CTF (Contrast Transfer Function) estimation was performed using CTFFIND (version 4.1). A total of 8375 particles were picked manually. Particles were extracted with a box size of 1440 pixels and then Fourier-cropped to 512 pixels for particle curation and initial model building. Iterative two-dimensional (2D) image classification was used to curate the data, and poorly aligned particles were removed from the final image stack. An Ab initio model was generated using stochastic gradient descent method without imposing any symmetry. The model was then aligned with its phase origin with an icosahedral symmetry using “relion_align_symmetry” program before being used as a reference map for 3D classification. The selected 3D class was selected for further refinement. The refined particle images were reextracted without rescaling and were curated using 2D classification without alignment. The final model was refined against the selected 5748 particles, supplying with a mask to remove the pixels within the inner sphere of a radius of 285 Å. The resolution was determined at 4.17 Å using gold standard Fourier Shell Correction (FSC) criteria (*44*). Particles were reextracted at a 1260-pixel box size to reduce Nyquist limitations. Refinement at this box size could not be run with GPU acceleration due to limitations in video card memory and therefore took careful consideration of memory requirements. Each iteration of refinement took approximately 3 days to finish while running on 9 MPI (Message Passing Interface) cores. Particles underwent CTF refinement with magnification anisotropy, which resulted in a 3.50 Å map after refinement. This was followed by further CTF correction, which improved the map resolution to approximately 3.32 Å. Bayesian polishing resulted in negligible improvements. Last, The Ewald sphere curvature was corrected to yield a final map with an average resolution of 3.07 Å as determined by RELION’s implementation of gold standard FSC (*45*) or 3.04 Å as determined by Phenix’s phenix.mtriage function. The resulting map was sharpened with DeepEMhancer using highRes, tightTarget, and wideTarget training models and required 2.5 days to run on 4 NVIDIA V100 GPUs.

### ChAdOx1 capsid model building

The amino acid sequence of the ChAdOx1 virus capsid proteins are described in the genome sequence of the source ChAdV-Y25 used to develop the ChAdOx1 vector (NC_017825.1). There are no differences between ChAdOx1 and Y25 in the capsid proteins.

The I-TASSER web server (*46*) or SWISS-MODEL (*47*) was used to build homology models of the various ChAdOx1 capsid proteins. Initially, the atomic models obtained from the web server were rigid body fitted to the capsid density map using ChimeraX (*48*), first by manual placement and then using the fit in map algorithm, beginning with the hexons, then the penton, pVIII, pIX, and pIIIa, respectively, until a full asymmetric unit had been assembled. After initial placement of the atomic model inside the density map, the model was fitted using the method molecular dynamics flexible fitting (MDFF) in explicit solvent (*49*, *50*). To avoid fitting of atomic models into adjacent density, additional protein models were added surrounding the asymmetric unit to occupy those regions of the density map. This “buffered” asymmetric unit was then subjected to another round of MDFF in explicit solvent to improve the quality of fit to the density map while accounting for protein-protein interactions between protein subunits. Models were then inspected, and loop regions with low-resolution density were manually modeled using ISOLDE (*51*). The model was further refined using symmetry restrained MDFF to address clashes between asymmetric units and further improve the local fits at the asymmetric unit edge (*52*). All MDFF simulations were performed in NAMD 2.14 (*53*) using the CHARMM36 (*54*) force field for protein, water, and ions at 300 K. The initial system for every MDFF simulation was prepared using the software Visual Molecular Dynamics (VMD) 1.9.3 (*55*). The potential energy function (*U*_EM_), obtained by converting from the EM density map was applied to the protein backbone during flexible fitting with a *g*-scale of 0.3. During flexible fitting, standard restraints were applied to maintain secondary structure, cis-peptide, and chirality in protein structures (*56*). The model was then further refined using ISOLDE as implemented in ChimeraX. Last, residues for which there was no signal were deleted from the model.

### Crystallization and structure determination of the ChAdOx1 Fiber knob protein

#### Production and purification of ChAdOx1 fiber knob protein

The method used to purify ChAdOx1 fiber knob protein is identical to that described for HAdV-D26 and HAdV-D48, previously. To summarize, SG13009 *Escherichia coli* containing the pREP-4 plasmid were transfected with pQE-30 expression vector containing the ChAdOx1 fiber knob transgene, located C-terminal to the 6His tag site, consisting of the 13 residues preceding the TLW motif to the terminal residue. These *E. coli* were selected in ampicillin (100 μg/ml) and kanamycin (50 μg/ml). They were cultured in 25 ml of LB with ampicillin (100 μg/ml) and kanamycin (50 μg/ml) overnight from glycerol stocks. Two liters of Terrific Broth (Terrific Broth, modified, Sigma-Aldrich) containing ampicillin (100 μg/ml) and kanamycin (50 μg/ml) was inoculated with the overnight culture and incubated at 37°C for 4 hours until it reached an optical density (OD) of 0.6 at λ570 nm. Once it reached the OD_0.6_, isopropyl-β-d-thiogalactopyranoside at a final concentration of 0.5 mM was added, and the culture was incubated for 18 hours at 21°C. Cells were harvested by centrifugation at 3000*g* for 10 min at 4°C, resuspended in lysis buffer [50 mM tris (pH 8.0), 300 mM NaCl, 1% (v/v) NP-40, lysozyme (1 mg/ml), 1 mM β-mercaptoethanol, and 10 mM imidazole], and incubated for 15 min shaking at room temperature. Lysate was then centrifuged at 30,000*g* for 20 min at 4°C and filtrated through 0.22-μm syringe filter (Milipore, Abingdon, UK). The filtered lysate was then loaded to 5-ml HisTrap FF nickel affinity chromatography column (GE Life Science) at 2.0 ml/min and washed with 15 ml into elution buffer A [50 mM tris-HCl or base (pH 8.0), 300 mM NaCl, and 1 mM β-mercaptoethanol]. Elution was done using a gradient rate of 20 min/ml from of buffer B [50 mM tris (pH 8.0), 300 mM NaCl, 1 mM β-mercaptoethanol, and 400 mM imidazole]. Collected fractions were concentrated by centrifugation using a Vivaspin 10,000 MWCO column (Sartorious, Goettingen, Germany) and analyzed by a SDS–polyacrylamide gel electrophoresis (SDS-PAGE) gel stained with Coomassie blue (correct bands are approximately 25 kDa). A second round of purification was performed using a GE 10/300 GL Increase Superdex 200 (GE Life Science) size exclusion chromatography at 0.5 ml/min and washed with 50 ml of Baker buffer [50 mM NaCl and 10 mM tris (pH 7.6)]. Fractions were then analyzed using an SDS-PAGE gel stained with Coomassie blue to check the purity and molecular weight.

#### Crystallization conditions

Final protein concentration was 13.5 mg/ml. For crystallization, the BCS and PACT Premier commercial crystallization screens (Molecular Dimensions) were used. Crystals were grown at 20°C in sitting drops containing 1:1 (v/v) ratio of protein to mother liquor. Microseeding was required for optimal crystal growth. Microseeding experiment was set up using Mosquito crystallization robot. Crystals appeared between 5 and 14 days.

Crystallization condition for solved structure was 0.1 M calcium chloride dihydrate, 0.1 M magnesium chloride hexahydrate, 0.1 M Pipes (pH 7.0), 22.5% (v/v) PEG (polyethylene glycol) smear medium, 0.2 M calcium chloride dihydrate, 0.1 M tris (pH 8.0), and 20% (w/v) PEG 6000.

#### Structure determination

Diffraction data were recorded on DIAMOND beamline DLS-I03, using GDA (Generic Data Acquisition) to control data collection. Automatic data reduction was completed with XDS and DIALS, and equivalents were scaled and merged with AIMLESS and TRUNCATE (*57*). The unique dataset was used in PHASER to solve the structure with molecular replacement, using a search model prepared by SWISS-MODEL on the basis of the structure of adenovirus C5 fiber knob protein, Protein Data Bank (PDB) entry 1KNB (*58*). Repeated cycles of graphics sessions in Coot (*59*) and refinement in REFMAC5 resulted in the final model presented in this manuscript. It became clear that the dataset suffered from twinning, which was automatically determined in REFMAC5 as one twin law, with a fraction of ~0.15. Details of data collection and refinement statistics are included in fig. S2A. Final coordinates are deposited in the worldwide PDB (wwPDB) as entry 7OP2.

### Modeling of fiber knob CAR interfaces

The preexisting structure of the HAdV-D37 fiber knob in complex with CAR D1 (PDB 2J12) (*19*) was used as a template by which to fit other fiber knob proteins, as previously described. The preexisting structure of HAdV-C5 (PDB 1KNB) (*58*) and HAdV-B35 (PDB 2QLK) (*60*) were aligned to the HAdV-D37 fiber knob in PyMOL using the “cealign” command, as was the fiber knob structure of ChAdOx1 (*61*). New models were saved containing the three CAR chains and one of the fitted chimeric fiber knob trimers. These homology models underwent 10,000 steps of energy minimization using a conjugate gradient and line search algorithm native to NAMD (*53*) and equilibrated by a short 2-ns molecular dynamics simulation. These models, seen in Fig. 2 (A to C) and fig. S3, were imaged, and binding interaction between the all three fiber knob and CAR protein-protein interfaces was scored at each frame of the MD trajectory using the Rosetta InterfaceAnalyzer tool (*62*, *63*).

### Determination of relative IC_50_ values of CAR and CD46 binding for Fiber knob proteins

Antibody binding inhibition assays were performed as previously described (*18*). CHO-CAR and CHO-BC1 cells were harvested, and 40,000 cells per well were transferred to a 96-well V-bottomed plate (Nunc, 249662). Cells were washed with cold PBS before seeding and kept on ice. Serial dilutions of recombinant soluble knob protein were made up in serum-free RPMI 1640 to give a final concentration range of 0.00001 to 100 μg per 105 cells. Recombinant fiber knob protein dilutions were added in triplicate to the cells and incubated on ice for 30 min. Unbound fiber knob protein was removed by washing twice in cold PBS, and primary CAR RmcB (Millipore, 05-644) or primary CD46 MEM-258 (Thermo Fisher Scientific, MA1-82140) antibody was added to bind the appropriate receptor. Primary antibody was removed after 1-hour incubation on ice, and cells were washed twice further in PBS and incubated on ice for 30 min with Alexa Fluor 488–labeled goat anti-mouse secondary antibody (Thermo Fisher Scientific, A-11001). Antibodies were diluted to a concentration of 2 μg/ml in PBS. Cells were washed and fixed using 4% paraformaldehyde and staining detected using BD Accuri C6 cytometer (BD Biosciences). Analysis was performed using FlowJo v10 (FlowJo, LLC) by sequential gating on cell population, singlets, and Alexa-488–positive cells compared to an unstained control. Total fluorescence intensity was determined as the Alexa-488–positive single-cell population multiplied by median fluorescence intensity, and IC_50_ curves were fitted by nonlinear regression using GraphPad Prism software to determine the IC_50_ concentrations.

### SPR binding assays

#### For fiber knob receptors

Binding analysis was performed using a BIAcore T200 equipped with a CM5 sensor chip. Approximately 2000 response units (RU) of CAR, DSG2, and CD46 were attached to the CM5 sensor chip using amine coupling, at a slow flow-rate of 10 μl/min. A blank flow cell was used as a negative control surface on flow cell 1. All measurements were performed at 25°C in PBS buffer (MilliporeSigma, UK) with 0.001% added P20 surfactant (GE Healthcare) at 30 μl/min. For binding analysis, the HAdV-C5, HAdV-B35, and ChAdOX fiber knob proteins were purified and flown over each sensor chip surface at a concentration of 0.5 M to confirm binding. To determine binding kinetics [on rate, *k*_a_ (1/ms); off rate, *k*_d_ (1/s)] and affinity [*K*_D_ (nM)], 1:2 serial dilutions were prepared for HAdV-C5K, HAdV-B35K, and ChAdOX fiber knob proteins and injected over CAR, DSG2, and CD46 immobilized on each sensor chip surface. The *K*_D_ and kinetic values were calculated assuming a 1:1 interaction using the BIAevaluation software.

#### For PF4 interactions

Assays were performed using a BIAcore T200, Cytiva (formerly GE Healthcare). The assay immobilization buffer was HBS-EP^+^ [10 mM HEPES, 150 mM NaCl, 3 mM EDTA, and 0.05% (v/v) Surfactant P20]. Virus at ~1 × 10^11^ VP/ml was diluted 1:5 in acetate 4.5 buffer and immobilized to a C1 sensor chip using a standard amine coupling protocol and a 15-min sample injection time. Typically, 400 to 500 RU of virus was immobilized. A reference sensor surface was created using the same amine coupling protocol but without the virus. Samples were injected with an association time of 60 to 120 s and a dissociation time of 60 to 120 s at a flow rate of 50 μl/min. The surface was regenerated with a 30-s injection of 25 mM NaOH at a flow rate of 50 μl/min. All sensorgram plots were subtracted from the reference flow cell and a buffer cycle to remove the nonspecific responses, bulk refractive index changes, and systematic instrument noise.

For the salt gradient experiments, PF4 protein (ChromaTec GmbH) was diluted to 2000 nM in HBS-EP^+^ with 1% bovine serum albumin (BSA) with various NaCl concentrations for the NaCl gradient experiments. For binding affinity measurements, PF4 was diluted to 3000 nM in PBS and 0.5% BSA. Two independent concentration series of PF4 was prepare using 1:3 dilutions. PF4-binding data were fit using the Biacore T200 Evaluation software using the steady-state affinity model.

For heparin competition injections, PF4 was prepared to 2000 nM, and heparin was prepared to 200,000 nM in PBS and 1% BSA. Samples were prepared as described: heparin alone (1:1 mix heparin stock and buffer), PF4 alone (1:1 mix of PF4 and buffer), and heparin and PF4 (1:1 mix of heparin stock and PF4 stock to a final molar ratio of 100:1).

### Sequence alignments

Sequence alignments were performed using the Clustal Omega algorithm as implemented in Expasy (*64*).

### Electrostatic surface calculations

The electrostatic effect from the icosahedral facet of ChAdOx1 on any point *r* = (*x*, *y*, *z*) of its surrounding environment is quantified by its electrostatic potential at *r*, which is denoted as *V*(*r*). This potential *V* was determined using the code, Adaptive Poisson-Boltzmann Solver (APBS) (*65*). APBS computed *V* using the charge distribution of ChAdOx1, the ion concentration of the environment surrounding ChAdOx1, and the permittivity of the environment. The computed *V* thus took into account the presence of counter ions in and the polarizability of the environment, which was a bulk of water molecules in our study. As the sign, + or −, of *V*(**r**) is highly correlated with the number of positive or negative charges around **r**, the potential for PF4 was also computed to visualize the overall charge distribution of PF4 upon its binding to ChAdOx1. The starting model for PF4 and PF4 in complex with fondaparinux was PDB 1RHP and 4R9W, respectively.

### BD simulation of the icosahedral facet in solution with PF4

Multireplica BD simulations were performed using the code, Atomic Resolution Brownian Dynamics (ARBD) (*66*). During the simulations, copies of PF4 were treated as rigid bodies diffusing in the neighborhood of the icosahedral facet of ChAdOx1 in solution.

The equation of motion (EOM) obeyed by each copy of PF4 for its diffusion in BD simulations is the overdamped Langevin dynamics. This EOM requires knowledge of the forces from the environment on PF4 and its damping coefficients with the environment. These coefficients, translational damping coefficients and rotational damping coefficients, were determined using the code, Hydropro (*67*).

The forces from the environment on PF4 consisted of two parts. The first part consisted of the random forces from thermal fluctuations. These random forces were automatically generated by ARBD during simulations on the basis of the system temperature *T*, which is 310 K here. The second part consisted of the electrostatic force and the van der Waals (VdW) force on PF4 from the icosahedral facet. The electrostatic force on PF4 with its center of mass at any point **r** was computed by ARBD automatically using the electrostatic potential *V* of the icosahedral facet and the charge distributions of PF4 around **r**. Similarly, we specify the VdW force between the icosahedral facet and PF4 using a potential, the Lennard-Jones (LJ) potential, denoted as *U* here. This potential *U* was computed using the code, VMD (*55*). LJ parameters needed for this computation were adopted from the Charmm36m force field (*68*).

The various potentials for the icosahedral facet and the damping coefficients for PF4 were feed into ARBD for multireplica BD simulations. Our work used 16 replicas of simulations in parallel. Each replica lasted for a simulation time of 2 μs and simulated 25 copies of PF4 diffusing around the icosahedral facet. During the simulations, these 25 copies of PF4 were only allowed to interact with the icosahedral facet and did not interact with one another. Thus, together, we performed 400 independent diffusion simulations with PF4. During each of these simulations, PF4 was allowed to bind to, or unbind, from the icosahedral facet. It is important to note that the residence time for each of these binding events is usually two to three order of magnitudes different compared with the real binding time. This is because the postbinding conformational changes of PF4, which often stabilize the interaction, are not sampled. Further, the simulations are performed in an implicit solvent environment, which is known to accelerate protein dynamics and diffusion, even in MD. Flexibility was simulated by applying a 3D normal distribution with an SD of 2 Å on each axis to each atom.

## Notes

View/request a protocol for this paper from *Bio-protocol*.
