# Supplementary material for: ChAdOx1 interacts with CAR and PF4 with implications for thrombosis with thrombocytopenia syndrome
Source: Sci Adv. 2021 Dec 1;7(49):eabl8213. doi: 10.1126/sciadv.abl8213 (PMC8635433; doi:10.1126/sciadv.abl8213)
Supplement: Supplementary file 1 — Figs. S1 to S10 [file sciadv.abl8213_sm.pdf]

Science Advances
AAAS

Supplementary Materials for

ChAdOx1 interacts with CAR and PF4 with implications for thrombosis with thrombocytopenia syndrome

Alexander T. Baker*, Ryan J. Boyd, Daipayan Sarkar, Alicia Teijeira-Crespo, Chun Kit Chan, Emily Bates, Kasim Waraich, John Vant, Eric Wilson, Chloe D. Truong, Magdalena Lipka-Lloyd, Petra Fromme, Josh Vermaas, Dewight Williams, LeeAnn Machiesky, Meike Heurich, Bolni M. Nagalo, Lynda Coughlan, Scott Umlauf, Po-Lin Chiu, Pierre J. Rizkallah, Taylor S. Cohen*, Alan L. Parker*, Abhishek Singharoy*, Mitesh J. Borad*

*Corresponding author. Email: baker.alexander@mayo.edu (A.T.B.); taylor.cohen@astrazeneca.com (T.S.C.); parkeral@cardiff.ac.uk (A.L.P.); asinghar@asu.edu (A.S.); borad.mitesh@mayo.edu (M.J.B.)

Published 1 December 2021, *Sci. Adv.* **7**, eabl8213 (2021)
DOI: 10.1126/sciadv.abl8213

This PDF file includes:

Figs. S1 to S10

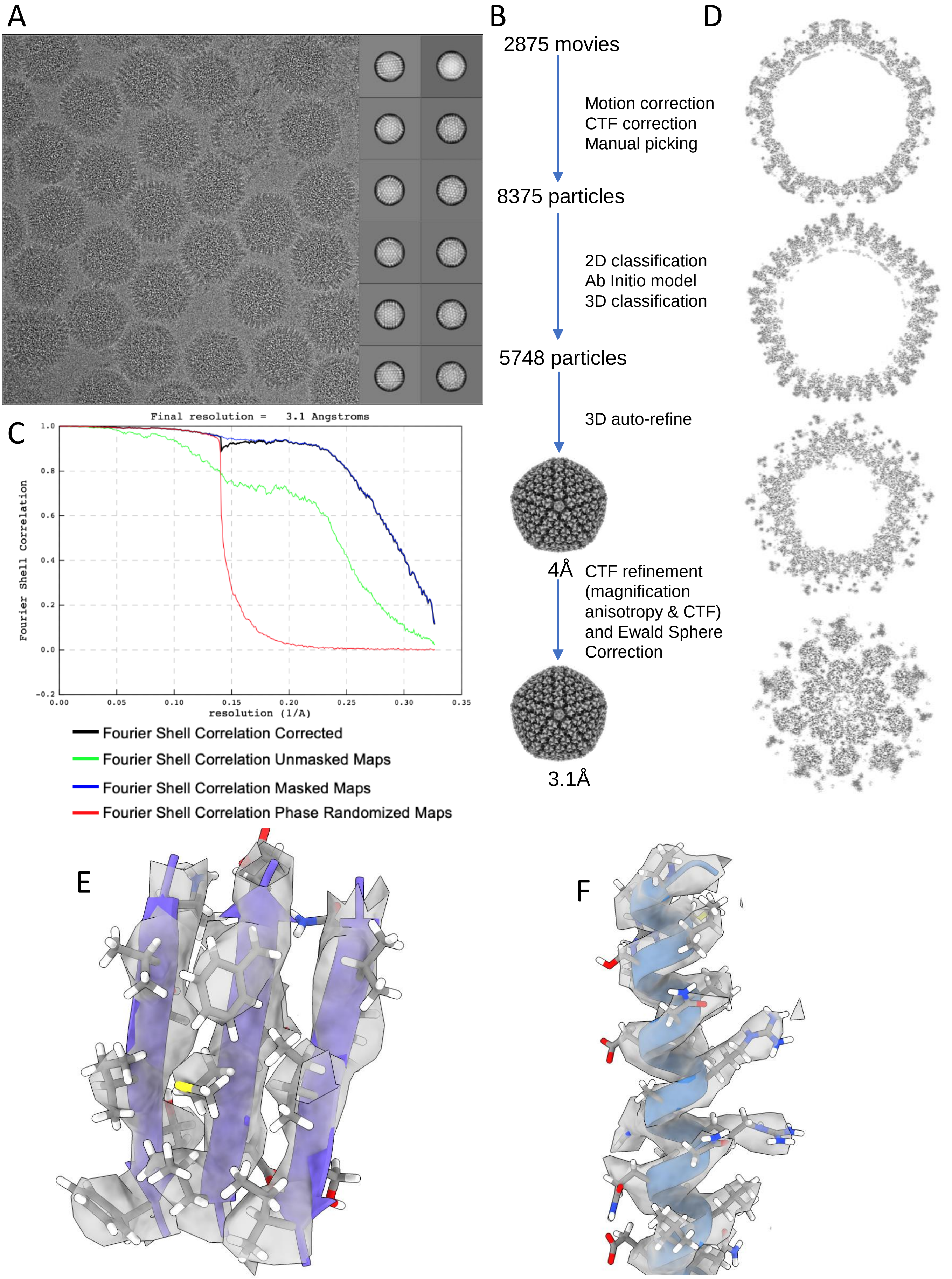

Fig.S1: **Validation statistics for CryoEM ChAdOx1 structure.** Particles were hand picked from micrographs and 2D classified (**A**). After classification and refinement (**B**) a 3.07Å volume was generated with acceptable Fourier shell correlation (**C**). Slices through the ChAdOx1 CryoEM volume show the localized resolution at the capsid interior has higher resolution information than the exterior which contains more flexible regions. An equatorial slice shows greater detail on the capsid interior, revealed in more detail by slices at points further along the 5-fold axis (**D**). Examples show hexon (**E**) and pVIII (**F**) model in density at contour level 0.015.

A

PDB Entry	7OP2
**Data Collection**	
Diamond Beamline	DLS-I03
Date	2021-04-24
Wavelength	0.97626
**Crystal Data (figures in brackets refer to outer resolution shell)**	
Crystallization Conditions	0.2 M CaCl2, 0.1 M Tris, pH 8.0, 20 % w/v PEG 6000
a,b,c (Å)	98.427, 112.26, 98.605
α=β=γ (°)	90.0, 92.61, 90.0
Space group	P 1 2_1 1
Resolution (Å)	1.59 – 74.03
Outer shell	1.59-1.62
R-merge (%)	27.3 (365.2)
R-pim (%)	8.0 (103.1)
R-meas (%)	28.5 (379.7)
CC1/2	0.996 (0.334)
I / σ(I)	5.5 (0.4)
Completeness (%)	100 (100)
Multiplicity	12.7 (13.5)
Total Measurements	3,364,401 (193,086)
Unique Reflections	286,462 (14,263)
Wilson B-factor($Å^2$)	14.6
**Refinement Statistics**	
Refined atoms	18,792
Protein atoms	17,316
Non-protein atoms	4
Water molecules	585
R-work reflections	271,539
R-free reflections	14,210
R-work/R-free (%)	22.4 / 24.4
**rms deviations (target in brackets)**	
Bond lengths (Å)	0.012 (0.013)
Bond Angles (°)	1.498 (1.656)
[1]Coordinate error	0.136
Mean B value ($Å^2$)	24.8
**Ramachandran Statistics (PDB Validation)**	
Favoured/allowed/Outliers	2143 / 84 / 4
%	96.0 / 4.0 / 0

* One crystal was used for determining each structure.

[1] Coordinate Estimated Standard Uncertainty in (Å), calculated based on maximum likelihood statistics.

B

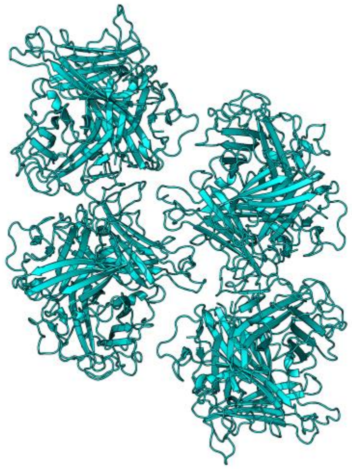

C

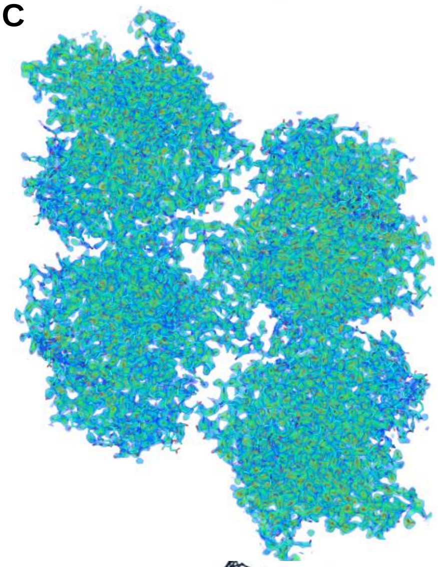

D

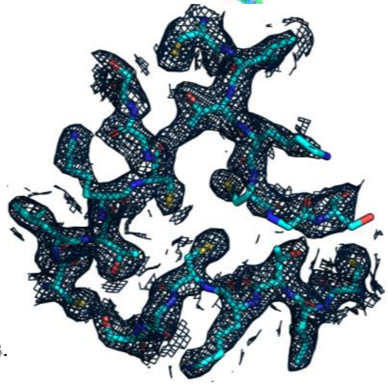

Fig.S2: Crystallization of ChAdOx1 fiber-knob protein results in 4 copies of the expected trimer per asymmetric unit and reveals side-chain locations. *Acceptable refinement statistics were achieved for the fiber-knob protein of ChAdOx1 (A). The crystal structure was solved with 12 copies of the monomer in the asymmetric unit, packing to form 3 trimeric biological assemblies (B). Density was sufficient to provide a complete structure in all copies (C, volume rendered in 0.5σ steps from red, 3.0σ, to dark blue), and was able to resolve side chain orientations reliably throughout the core fold (D, mesh shown at σ=1.0).*

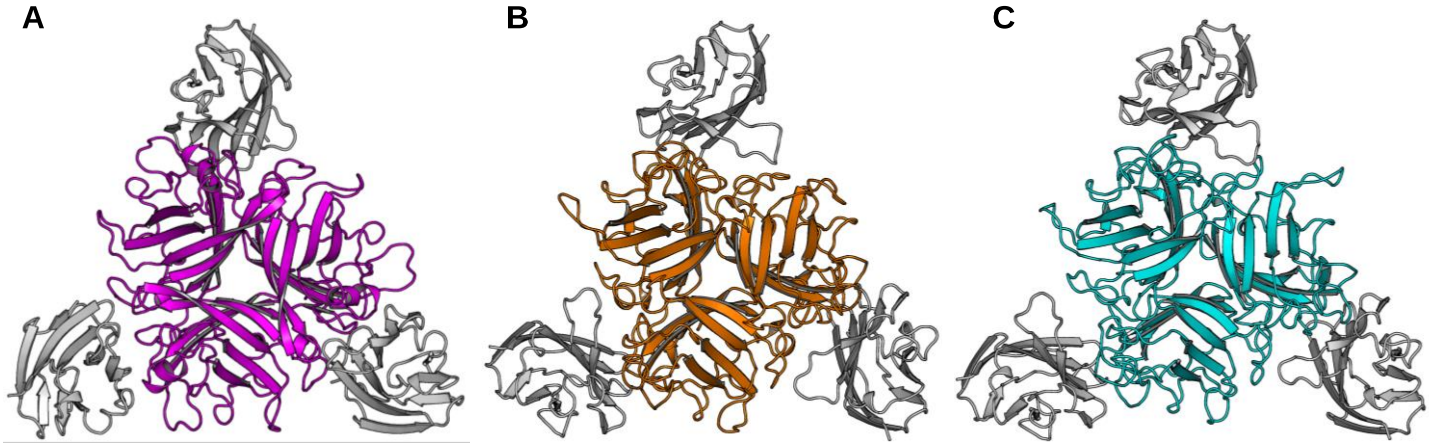

Fig.S3: Homology models of adenovirus fiber-knobs with CAR. *Using PDB 2J12 as a template the fiber-knob structures of HAdV-B35 (A, purple), HAdV-C5 (B, orange), and ChAdOx1 (C, cyan) were aligned with CAR (grey) in a potential binding pose and equilibrated by molecular dynamics.*

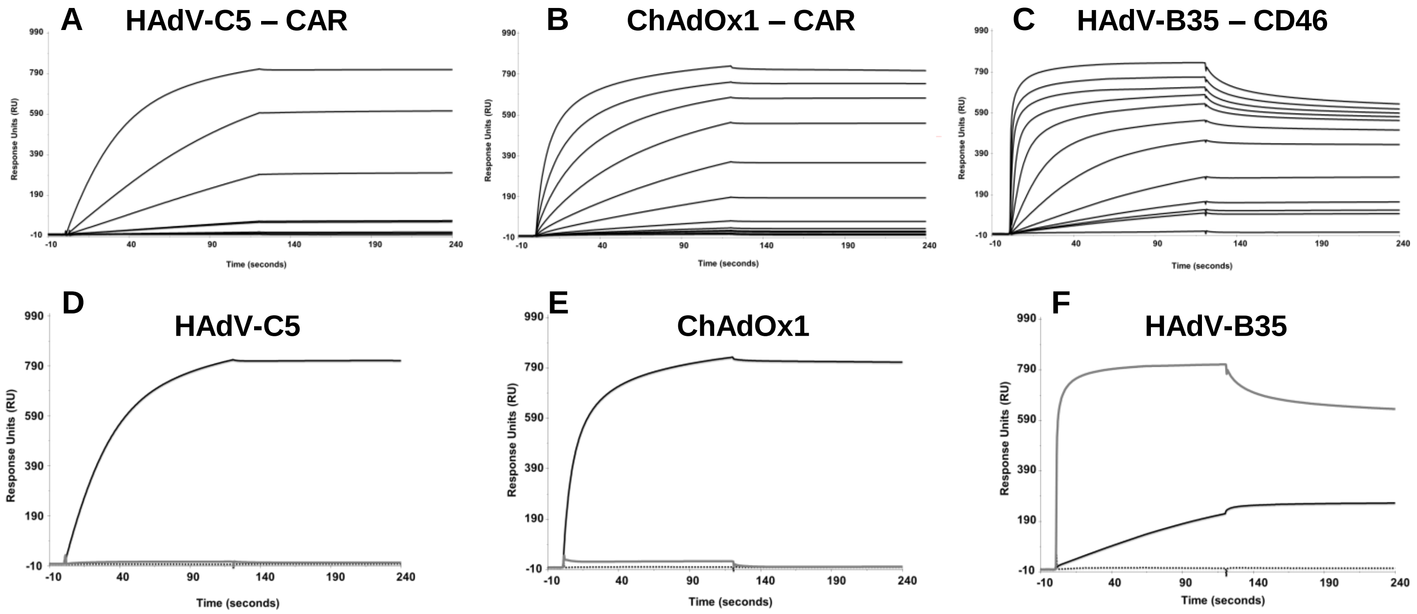

Fig.S4: Surface plasmon resonance traces show ChAdOx1 fiber-knob binds to CAR with high affinity but not CD46 or desmoglein 2. *Traces are shown as resonance units (RU) over time (seconds). Serial titration SPR shows HAdV-C5 (Titration=2.5-160nM) binds to CAR (KD=0.06±0.02nM,* ***A****) as does ChAdOx1 (Titration=2.5-2560nM, KD=7.16±1.92nM,* ***B****). HAdV-B35 (Titration=2.5-2560nM) binds to CD46 (KD=4.38±1.95nM,* ***C****). Further SPR experiments show HAdV-C5K (160nM) binds CAR (black), but not DSG2 (black, dashed) nor CD46 (grey,* ***D****). ChAdOX (2560nM) binds CAR (black), but not to DSG2 (black, dashed) and weakly interacts with CD46 (grey,* ***E****). HAdV-B35K (2560nM) binds CD46 (grey) and to a lesser degree to CAR (black) but not DSG2 (black dashed,* ***F****).*

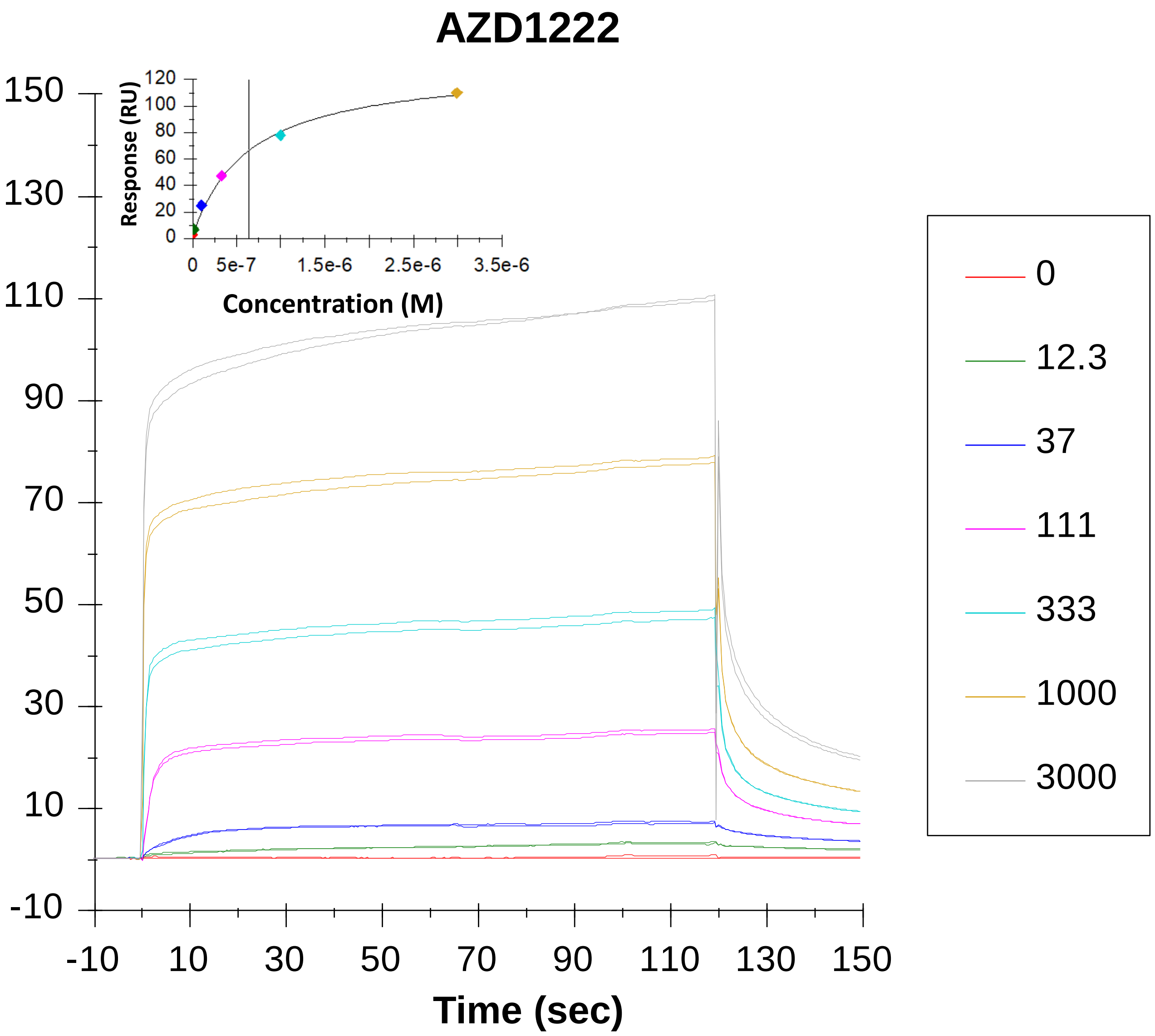

Fig.S5: The ChAdOx1 nCoV-19 vaccine preparation (AZD1222) binds to PF4 with high affinity. *Serial titration SPR at the indicated concentrations (nM, see legend) shows the ChAdOx1 nCoV-19 vaccine preparation binds to PF4 with affinity (*K_D= 514 ± 40 nM*) comparable to that of CsCl purified adenoviruses as determined by the steady state model (inset figure).*

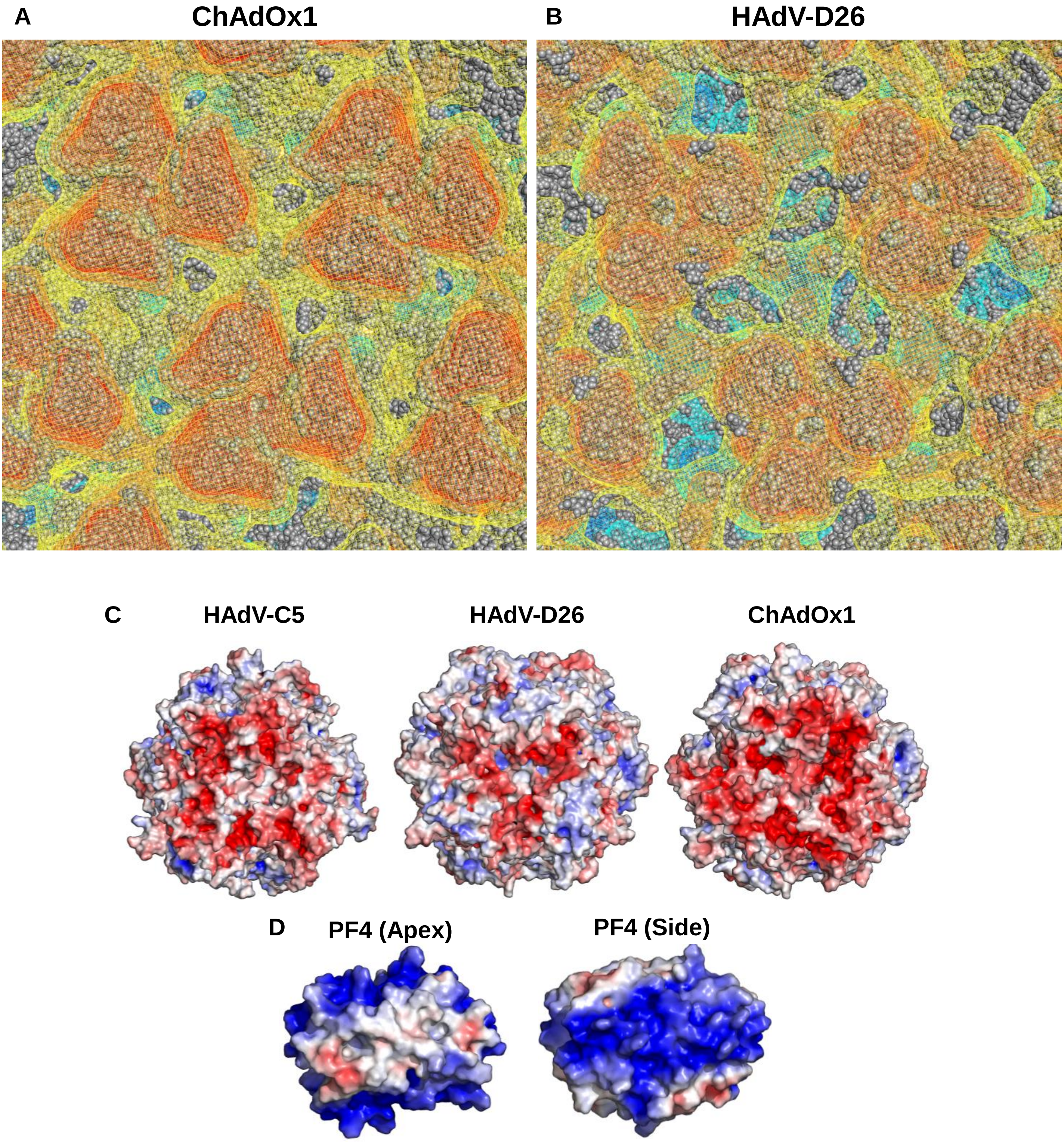

Fig.S6: ChAdOx1 is strongly electronegative, the opposite of PF4. *Close-up inspection of the electrostatic surface of ChAdOx1 (**A**) and HAdV-D26 (**B**) shows the strong electronegative potential emanating from the hexon apexes, with regions of electropositive potential in the space between hexons. The electronegative potential is substantily stronger in ChAdOx1, while the regions of electropositive potential are stronger in HAdV-D26. Visualisation in individual hexons shows how the apext of the trimer is electronegative around the apex of the 3 fold axis, and that the charge is strongest in ChAdOx1, followed by HAdV-C5, and weakest in HAdV-D26, with HAdV-D26 showing the strongest electropositive charges in the lateral regions (**C**). This contrasts with PF4, which has a strongly overall electropositive charge (**D**). Continuum electrostatic calculations are shown as a mesh from -0.5K_BT (yellow), -1.0K_BT (orange), -1.5K_BT (red), 0.5K_BT (cyan), 1.0K_BT (blue), and 1.5K_BT (dark blue). APBS is visualized on a +/-5.0eV ramp from blue to red.*

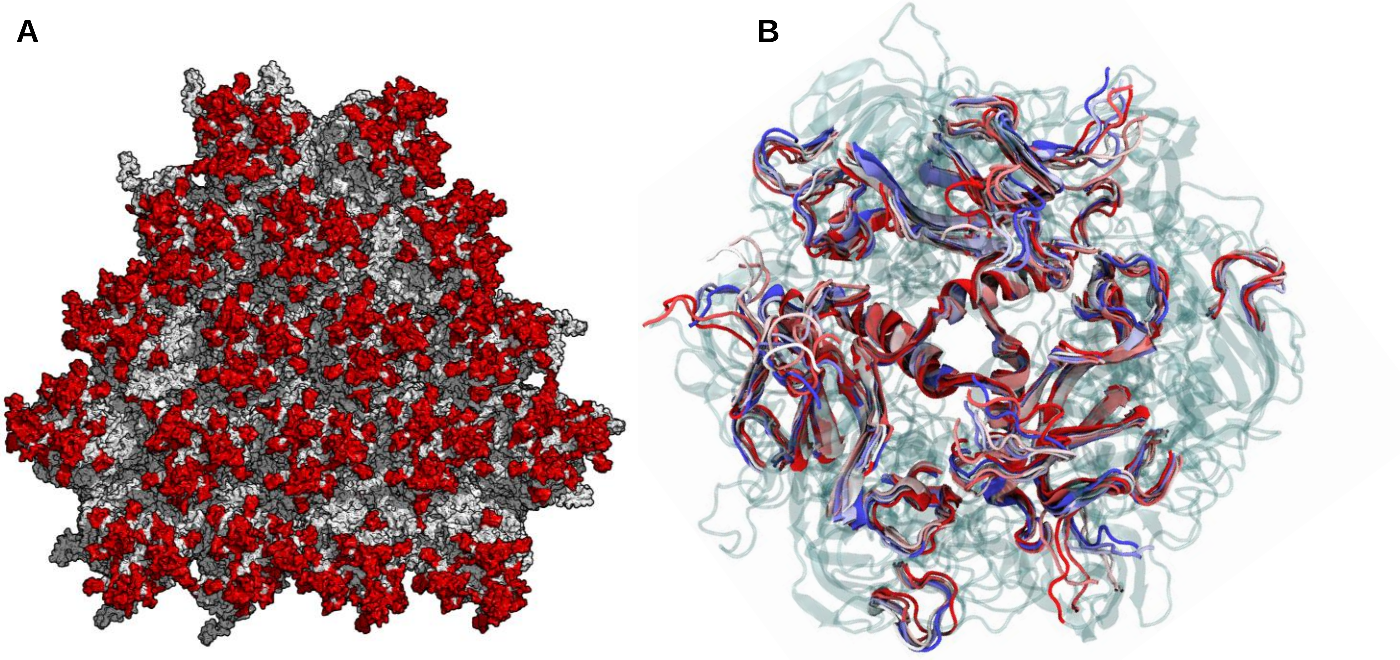

Fig.S7: The ChAdOx1 hexon HVRs face into the space between hexons and are highly flexible. *The hyper variable regions of the ChAdOx1 hexons (red) cluster about the apex and present into the space between hexons (**A**). Molecular dynamics simulations demonstrate that the HVRs are highly flexible (**B**, HVR positions are shown in full color).*

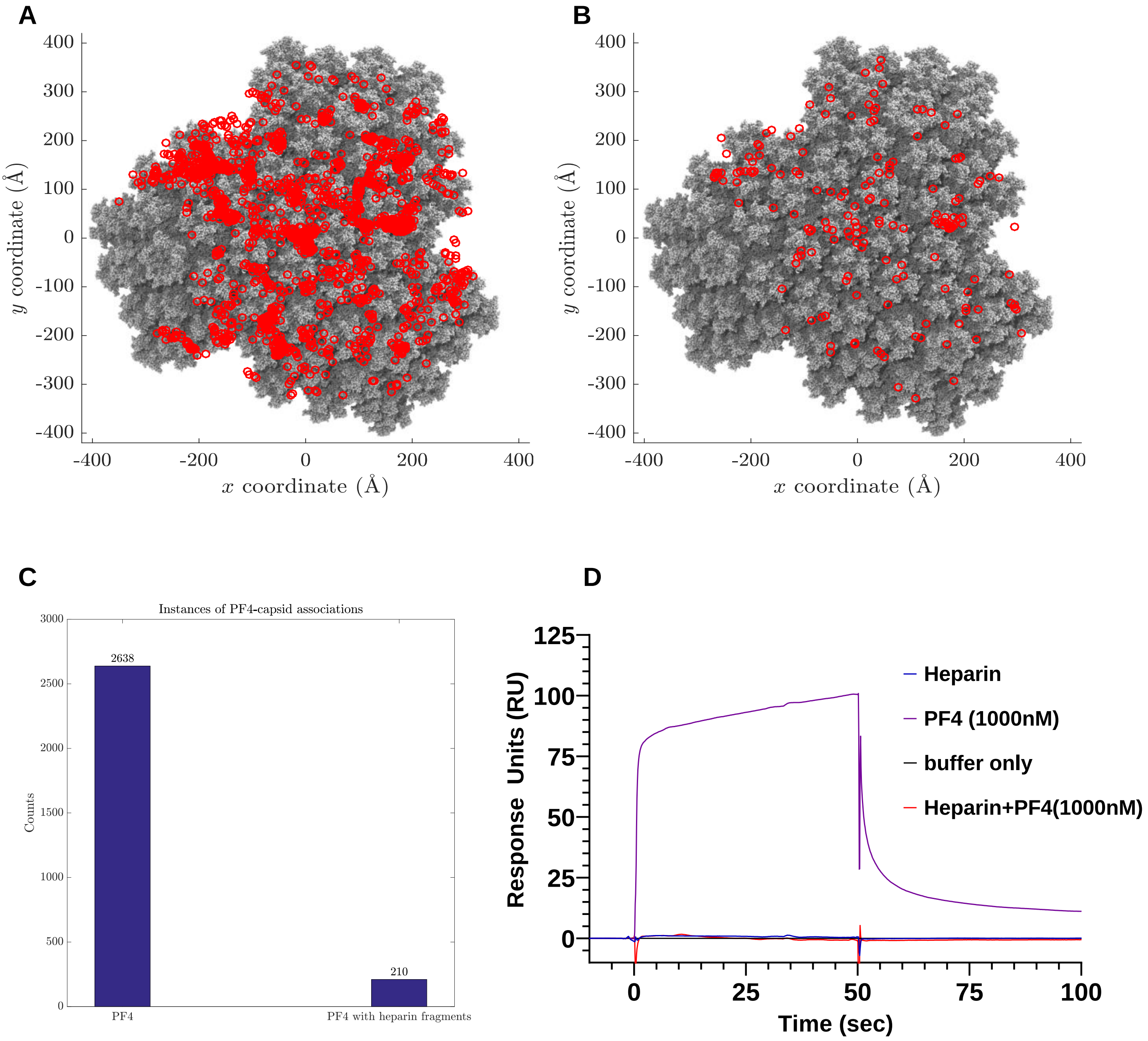

Fig.S8: The ChAdOx1 nCoV-19 vaccine preparation binds to PF4 with high affinity, but this interaction is weakened by the presence of heparin. *Brownian dynamics simulations show frequent interactions (red spots) between the PF4 tetramer and the ChAdOx1 surface (grey) (A). Similar simulations performed with the PF4-Fondaparinux (PDB 4R9W) showed the frequency of interactions reduced (B) by 12.56-fold (C). SPR shows that PF4 binds to the ChAdOx1 nCoV-19 vaccine preparation with high affinity, but when PF4 is preincubated with Heparin this affinity is drastically reduced (D).*

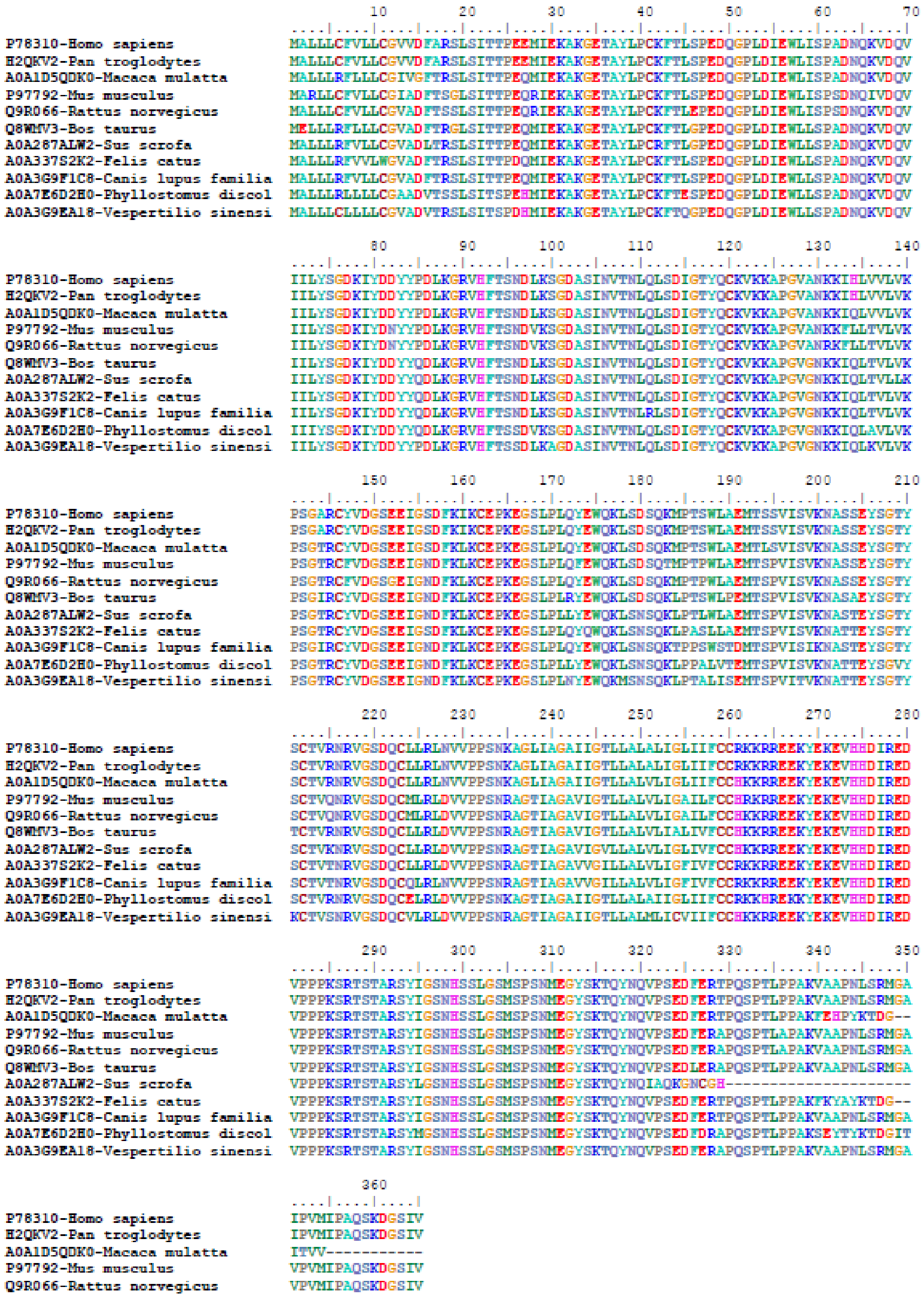

Fig.S9: CAR is a highly conserved protein across a range of scientifically important, domestic, and agriculturally significant species. *Humans (homo sapiens) and Chimpanzees (Pan Troglodytes) share a 100% sequence identity for their canonical CAR isoform. Sequences in this alignment taken from the indicted UniProt accession numbers.*

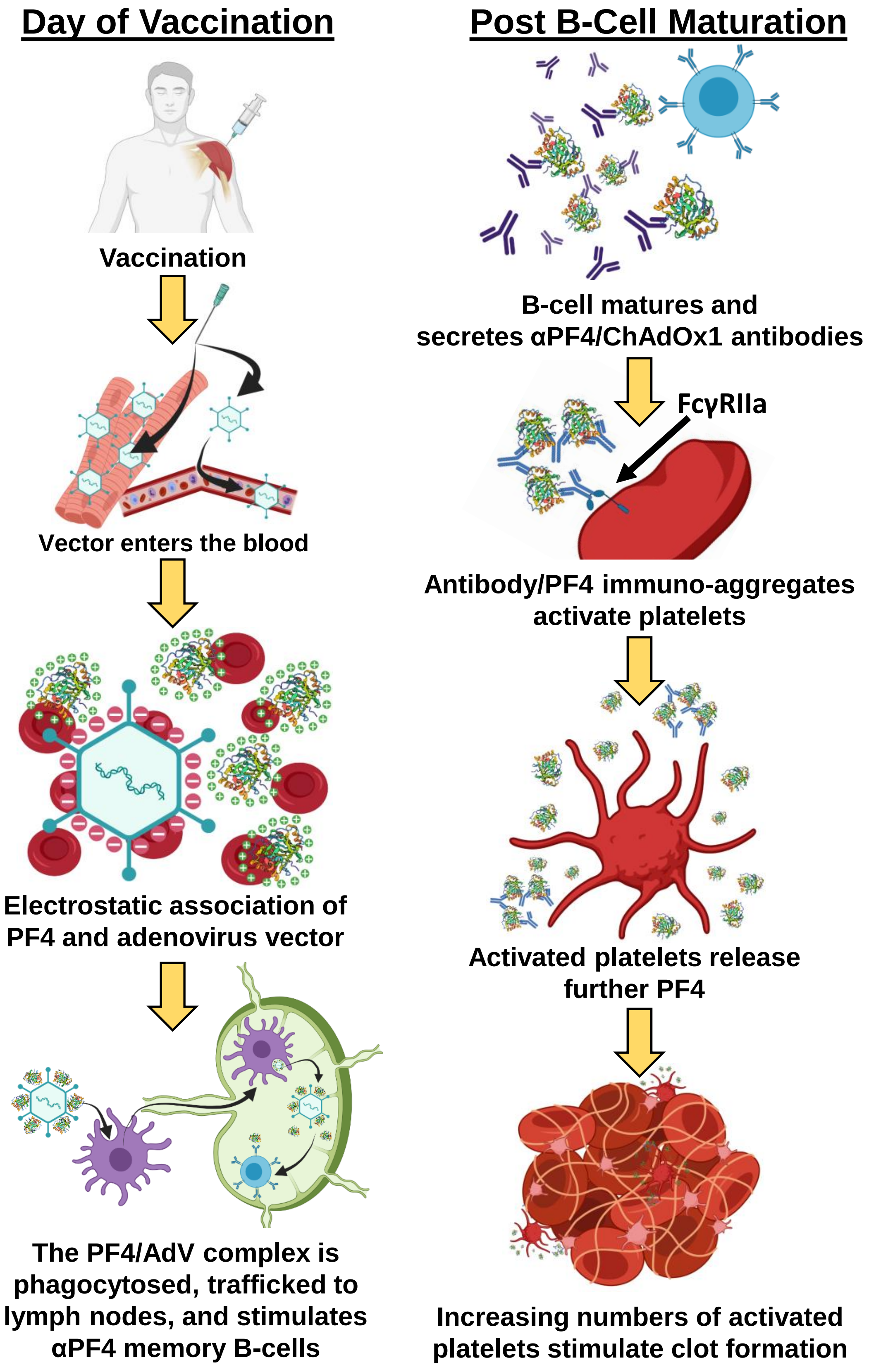

Fig.S10: Cartoon representation of a proposed mechanism by which ChAdOx1 association with PF4 might result in thrombotic thrombocytopenic syndrome. *Following intramuscular vaccination with ChAdOx1 nCoV-19 small quantities of viral vector may enter the blood where they could interact with PF4 and form a complex through the mechanisms described in this study. This complex can then be taken up by monocytes and transported to the lymph nodes where it may stimulate the proliferation of pre-existing PF4 specific B-cells. After maturation of these B-cells, >5 days later, αPF4 IgG will be secreted which can form aggregates with PF4 circulating in the blood. These aggregates can stimulate activation of platelets by binding to FcγRIIa, stimulating further PF4 release. This could trigger a positive feedback loop culminating in clot formation and NETs, as in HITT.*
